# Macrophage Migration Inhibitory Factor Antagonist Blocks the Development of Endometriosis In Vivo

**DOI:** 10.1371/journal.pone.0037264

**Published:** 2012-05-23

**Authors:** Khaled Khoufache, Sylvie Bazin, Karine Girard, Julie Guillemette, Marie-Christine Roy, Jean-Pierre Verreault, Yousef Al-Abed, Warren Foster, Ali Akoum

**Affiliations:** 1 Endocrinologie de la Reproduction, Centre de Recherche, Hôpital Saint-François d’Assise, CHUQ, Quebec City, Québec, Canada; 2 Département d’obstétrique et gynécologie, Faculté de médecine, Université Laval, Quebec City, Québec, Canada; 3 The Feinstein Institute for Medical Research, Manhasset, New York, United States of America; 4 Department of Obstetrics & Gynecology, McMaster University, Hamilton, Ontario, Canada; Université de Technologie de Compiègne, France

## Abstract

Endometriosis, a disease of reproductive age women, is a major cause of infertility, menstrual disorders and pelvic pain. Little is known about its etiopathology, but chronic pelvic inflammation is a common feature in affected women. Beside symptomatic treatment of endometriosis-associated pain, only two main suboptimal therapeutic approaches (hormonal and invasive surgery) are generally recommended to patients and no specific targeted treatment is available. Our studies led to the detection of a marked increase in the expression of macrophage migration inhibitory factor (MIF) in the eutopic endometrium, the peripheral blood and the peritoneal fluid of women with endometriosis, and in early, vascularized and active endometriotic lesions. Herein, we developed a treatment model of endometriosis, where human endometrial tissue was first allowed to implant into the peritoneal cavity of nude mice, to assess *in vivo* the effect of a specific antagonist of MIF (ISO-1) on the progression of endometriosis and evaluate its efficacy as a potential therapeutic tool. Administration of ISO-1 led to a significant decline of the number, size and *in situ* dissemination of endometriotic lesions. We further showed that ISO-1 may act by significantly inhibiting cell adhesion, tissue remodeling, angiogenesis and inflammation as well as by altering the balance of pro- and anti-apoptotic factors. Actually, mice treatment with ISO-1 significantly reduced the expression of cell adhesion receptors αv and ß3 integrins (P<0.05), matrix metalloproteinases (MMP) 2 and 9 (P<0.05), vascular endothelial cell growth factor (VEGF) (P<0.01), interleukin 8 (IL8) (P<0.05), cyclooxygenease (COX)2 (P<0.001) and the anti-apoptotic protein Bcl2 (P<0.01), but significantly induced the expression of Bax (P<0.05), a potent pro-apoptotic protein. These data provide evidence that specific inhibition of MIF alters endometriotic tissue growth and progression *in vivo* and may represent a promising potential therapeutic avenue.

## Introduction

Endometriosis, a gynecological complication characterized by extra-uterine localization of endometrial tissue, mainly in on pelvic organs, affects 5 to 10% of reproduction age women [1]. Its diagnosis remains very difficult, but a positive diagnosis is generally associated with pelvic pain (60%), dysmenorrhea (30%), dyspareunia (36%) and infertility (50%) [2]. Endometriosis is hormone-dependent and genetic and environmental factors may play a role in its development [3], [4], [5]. Beside symptomatic treatment of endometriosis-associated pain, only two main suboptimall therapeutic approaches in particular hormonal and invasive surgical [6], [7] are generally recommended to patients and no specific targeted treatment is available. Chronic pelvic inflammation is a hallmark of endometriosis pathophysiology. Evidence available to date indicates that immune and inflammatory factors, whether they are released by immune or peritoneal, endometrial and endometriotic cells, may play a critical role in the ectopic survival, implantation and growth of endometrial tissue [1], [8], [9], [10], [11]. Curiously, instead of eliminating misplaced endometrial cells, immune cells like macrophages are more activated in women with endometriosis and release factors that may exacerbate inflammation and facilitate endometrial tissue adhesion, invasion and growth within the host tissue [9], [12], [13], [14], [15], [16].

Our previous studies showed a marked increase in macrophage migration inhibitory factor (MIF) in eutopic endometrial tissue of women with endometriosis, which varied according to the disease’s stage and major symptoms [17]. We further found a significant elevation in the circulating [18] and local peritoneal [11] levels of MIF and an increased expression of this factor in early, vascularized and most active endometriotic lesions [19]. MIF was also overproduced by activated peritoneal macrophages of women with endometriosis. The available literature supports our findings [12], [20], [21], [22]. Initially, MIF was defined as a cytokine that inhibits macrophage migration [23]. But today, MIF is known as an important regulator of the host immune system that promotes the pro-inflammatory functions of immune cells [24], [25]. In addition, MIF has been shown to be implicated in angiogenesis, tumorigenesis, as well as in many inflammatory and autoimmune diseases [26], [27], [28], [29], [30], [31], [32], [33], [34], [35], [36]. Our previous studies further showed the capability of MIF to stimulate inflammation and favor angiogenesis in vitro and in vivo [28], [29], [31], [37]. Based on these findings, we hypothesize that MIF may and via different direct and indirect mechanisms play an important role in the development of endometriosis. The present study was therefore designed to evaluate the efficacy of a specific MIF inhibitor called ISO-1 as a potential treatment for endometriosis using an *in vivo* model of endometriosis. ISO-1 or (S,R) 3-(4-hydroxyphenyl)-4,5-dihydro-5-isoxazole acetic methyl ester) is defined as a highly specific inhibitor to the catalytic site of MIF [38]. Our data showed that treatment with ISO-1 leads to a significant regression of established ectopic endometrial implants and a marked down-regulation of angiogenic, tissue remodeling and survival factors, and may represent a promising approach for the treatment of peritoneal endometriosis.

## Materials and Methods

### Animals

Five-seven weeks old female athymic nude mice (Harlan, USA) were used in this investigation. The animals were housed (two/cage) under laminar-flow (HEPA)-filtered hoods in rooms maintained at 28°C with a 12:12-hour light-dark cycle. Housing material, food and water were sterilized before use. This study was approved by the “Comité de protection des animaux du CHUQ” (permit ID: 2009068-3). *In vivo* experiments were performed according to the Canadian committee of animal’s protection (CPA) rules.

Endometrial biopsies were obtained from four patients (mean age ± SD, 41.5±5.2 years) undergoing surgical explorative laparoscopy or hysterectomy for fibroma, myoma, bleeding and infertility problems. These patients signed an informed consent and accepted to participate to this research project, which was approved by Saint-François d’Assise Hospital ethics committee on human research (Laval University, Québec, Canada) (project ID: 5-00-12-07). Included women did not have endometriosis and were not receiving anti-inflammatory or hormonal medication for at least 3 months before surgery. Biopsies were collected using a Pipelle de Cornier (Prodimed, Neuilly-en-Tchelle, France) during the proliferative phase (d2-d13) of the menstrual cycle. The menstrual cycle phase was confirmed by histology [39]. Tissue samples were placed in cold sterile Hank’s balanced salt solution containing 100 IU/ml penicillin, 100 µg/ml streptomycin and 0.25 mg/ml amphotericin B (Invitrogen Life Technologies, Burlington, ON, Canada) and immediately transported to the laboratory. A small sample of each tissue was fixed at 4°C in 10% formalin and processed to paraffin for histological analysis and the remaining tissue was immediately used for *in vivo* studies.

### *In Vivo* Model of Endometriosis

**Figure 1:**
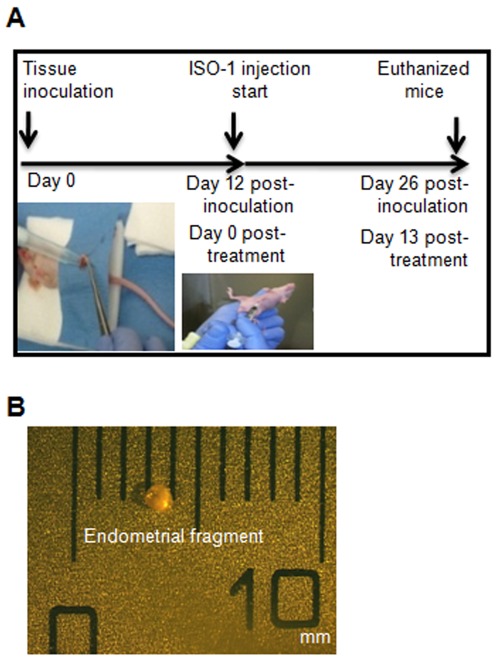
Schematic illustration of the experimental design. A) Human endometrial tissue was inoculated into the peritoneal cavity of mice using a micropipette and left for 12 days before starting treatment. ISO-1 was then intra-peritoneally administered on a daily basis for 14 consecutive days before euthanizing the animals. B) Size of injected endometrial fragments.

Endometrial tissue was washed twice with in phosphate buffered saline (PBS), dissected into thirteen small cubes (∼1×1 mm^3^) per mouse and labeled with carboxyfluorescein diacetate, succinimidyl ester (CFDA-SE) (8.10^−6^ M) (Invitrogen, Burlington, ON, Canada) during 20 min at room temperature. Tissue fragments were then washed twice in PBS and examined under a fluorescence stereomicroscope to confirm labeling. Animals were given buprenorphine (1.68g/mouse) by intradermal injection for analgesia, then anesthetized with a mixture of oxygen (1.5 l) and isoflurane® (3–4%) (Abbot Laboratories, Saint-Laurent, Quebec, Canada). The model mimics endometriosis in nude mice by inoculating endometrial tissue into the peritoneal cavity. To access the peritoneal cavity, we made a small incision (1 cm) in the cutaneous tissue and the underneath peritoneal tissue. Thirteen CFDA-labelled endometrial tissue fragments in 0.1 ml PBS were then injected into the peritoneal cavity through this opening using micropipette as shown in Figure 1A. The peritoneal incision was sutured with coated NB (polyglactin 910) sutures (Ethicon Johnson & Johnson, Ontario, Canada) and the cutaneous incision was closed with MikRon autoclip 9 mm (Clay Adam Brand, Sparks, USA). Inoculated mice were monitored daily for comfort, survival and body weight until day 12 post-inoculation. Beginning on day 12, animals received a daily intra-peritoneal injection (100 µl) of sterile PBS used as vehicle (control group, n = 8) or PBS containing ISO-1 (4 mg/kg) (n = 8) [38]. Injections were performed until day 25 post-inoculation. Animals were monitored daily for weight and survival rate (Figure 1A). Fourteen days post-treatment, animals were anesthetized with isoflurane/O_2_ (3–4%), then euthanized in an atmosphere saturated by CO_2_ (10 l/min) and the abdominal cavity of each mouse was explored using a fluorescence stereomicroscope for *in situ* identification, localization, enumeration and measure of endometriotic lesions using a caliper precision instrument (Figure 1B). Lesions were photographed *in situ* using a color CCD HD camera (DAGE-MTI, Michigan City, IN, USA). Finally, lesions were removed and stored at −80°C for molecular analysis or fixed in cold formaldehyde (10% in PBS) and embedded in paraffin for histological studies.

RNA extraction and quantitative real-time polymerase chain reaction (qRT-PCR).

Using the standard Trizol® protocol (Invitrogen) as previously reported [28], RNA was extracted separately from each surface and deep implant. qRT-PCR was performed using an ABI 7000 Thermal Cycler (Applied Biosystems, Foster City, CA). Each standard PCR reaction contained 2 µl reverse transcriptase (RT) product, 0.5 µl of each primer (final concentration, 0.1 mm), 12.5 µl SYBR Green PCR Master Mix (Invitrogen) consisting of *Taq* DNA polymerase reaction buffer, deoxynucleotide triphosphate mix, SYBR green I, MgCl_2_, and *Taq* DNA polymerase. The conditions of reaction and the listing of primers are reported in the Table 1. Primers were designed with Primer Premier 5 software to cross intron-exon boundaries. All samples were tested in duplicate and for each reaction; negative controls without RNA and without reverse transcriptase were added.

**Table 1:** List of PCR primers.

Gene	Primers	Tm	GeneBank accession #
Bax	F-TCAACTGGGGCCGGGTTGTC	60°C	NM_004324.3
	R-CCTGGTCTTGGATCCAGCC		
Bcl2	F-ACATGCTCGCCCAGCCAC	60°C	NM_000657.2
	R-TGCACATGGGGCATCTTGG		
COX2	F-TCAACTGGGGCCGGGTTGTC	60°C	NM_000963
	R-AAAACTGATGCGTGAAGTGCTG		
GAPDH	F- CAGGGCTGCTTTTAACTCTGG	60°C	NM_002046.3
	R-TGGGTGGAATCATATTGGAACA		
IL8	F-TGGCAGCCTTCCTGATTT	60°C	NM_000584.2
	R-AGGTTTGGAGTATGTCTTTATGC		
Integrin αv	F-GGAGCAATTCGACGAGCACT	60°C	NM_001144999.1
	R-TTCATCCCGCAGATACGCTA		
Integrin β3	F-TGACGAAAATACCTGCAACCG	60°C	NM_000212.2
	R-GCATCCTTGCCAGTGTCC		
MMP2	F-TTGACGGTAAGGACGGACTC	60°C	NM_001127891.1
	R-ACTTGCAGTACTCCCCATCG		
MMP-9	F-TTGACAGCGACAAGAAGTGG	60°C	NM_004994.2
	R-CCCTCAGTGAAGCGGTACAT		
TIMP1	F-AAGGCTCTGAAAAGGGCTTC	60°C	NM_003254.2
	R-GAAAGATGGGAGTGGGAACA		
VEGF	F-GCTCTACCTCCACCATGCCA	60°C	NM_001171630.1
	R-CACCACTTCGTGATGATTCTG		

### Histological Analysis

Four-µm sections of tissue were rehydrated and stained with hematoxylin and eosin (H & E) according to a standard procedure [40].

### Statistical Analysis

Statistical tests used in this study were the Kruskal-Wallis for the analysis of the evolution of the body weight during tissue inoculation and treatment, the Mann-Whitney for the number and volume of lesions per mouse and qRT-PCR data, which followed non-parametric distributions, and student t-test for the number and volume of lesions per tissue, which were normally distributed. Results were expressed as mean ± SEM. All statistical analyses were performed using GraphPad Prism software (San Diego, CA, USA). Differences were considered as statistically significant for P<0.05.

## Results

### Establishment of Experimentally-induced Endometriosis

**Figure 2:**
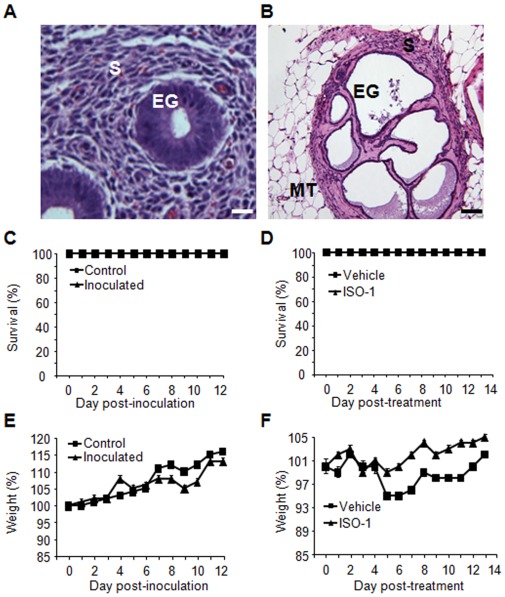
Histological examination of human endometrial implants in nude mice two weeks after inoculation of endometrial tissue. A) Initial endometrial tissue before inoculation into mice. B) Endometrial implant 12 days following tissue inoculation showing epithelial glands (EG) surrounded by endometrial stromal cells (S) and mouse tissue (MT). Hematoxylin-eosin staining; scale bar, 20 µm. C and D) Effect of endometrial tissue inoculation and treatment with ISO-1 on the survival rate of animals. E and F) Effect of endometrial tissue inoculation and treatment with ISO-1 on the body weight of animals. The Figure summarizes results obtained from 7 mice treated with ISO-1 (4 mg/kg) and 8 control mice treated with vehicle.

In this model, human endometrial tissue was allowed to implant and establish in nude mice before starting treatment. Histological analysis of implants prior to any treatment showed a well-preserved endometrial structure comparable to that of initial endometrial tissue, with glands surrounded by stroma, and distinguishable from the underneath murine host tissue. However, endometriotic lesions showed more dilated glands, which might be due to accumulation of glandular cell secretions (Figure 2, A and B). Induction of endometriosis had no perceptible effect on the health of these animals. All mice survived (Figure 2C) and no body weight difference between tissue-inoculated and control mice was observed (Figure 2E). The use of CFDA-SE fluorochrome allowed us to easily identify endometriotic lesions, and 60±2.5% of endometrial tissues initially injected per mouse were collected.

### Impact of the Treatment of Mice with ISO-1

#### Survival and weight of animals

All mice survived after treatment with 4 mg/kg ISO-1 (Figure 2D). On the other hand, five days post-treatment, animals exposed to vehicle showed some body weight loss estimated at 6.0±1.8%, which, however, was not significant as compared to the initial weight and recovered at the end of treatment. Animals exposed to ISO-1 showed no significant weight loss of gain (Figure 2F).

#### Number, size and histology of lesions

**Figure 3:**
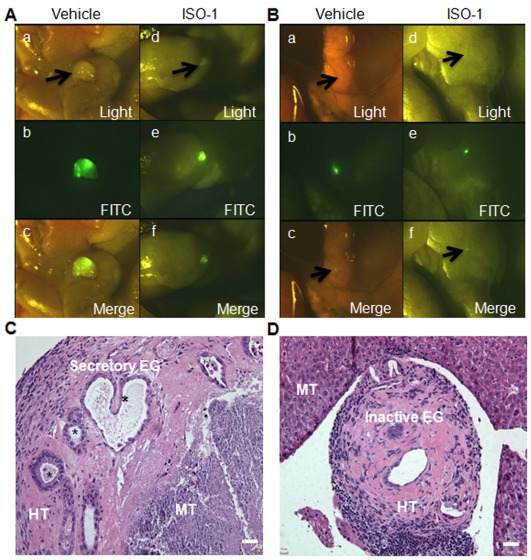
Stereomicroscopic observation and histological examination of endometrial implants. A and B) Surface and deep implants, respectively, were observed at sacrifice by fluorescence stereomicroscopy in mice treated with vehicle (a, b, c) or ISO-1 (4 mg/kg) (d, e, f). Implants, as observed by optic light (a, d) or epi-fluorescence (b, e). Merged images are shown (c, f). C and D) Histological examination of endometrial implants from control mice treated with vehicle or 4 mg/kg ISO-1, respectively. EG, endometrial gland; HT, human tissue; MT, mouse tissue; scale bar, 10 µm.

Fourteen days post-treatment, mice were euthanized and macroscopic exploration was immediately performed using a stereomicroscope equipped with a fluorescein isothiocyanate (FITC) filter. On gross morphological examination, mice treated with ISO-1 had a minimal sign of disease compared with control mice treated with the vehicle alone, which clearly had more, larger and well-defined endometriotic lesions (Figure 3A). Furthermore, deep infiltrating endometriotic lesions, which were only observable by fluorescence, were identified. These lesions were smaller in mice treated with ISO-1 than in control mice (Figure 3B). Histological evaluation of the harvested lesions showed a human endometrial tissue, which, in control mice, closely adhered to the host mouse tissue and exhibited many well-defined, secretory and active endometrial glands (Figure 3C). However, in mice treated with ISO-1, endometrial tissue was detached from the host tissue and showed essentially small, degenerate and most probably inactive glandular structures (Figure 3D).

#### Tissue invasiveness and dissemination

**Figure 4:**
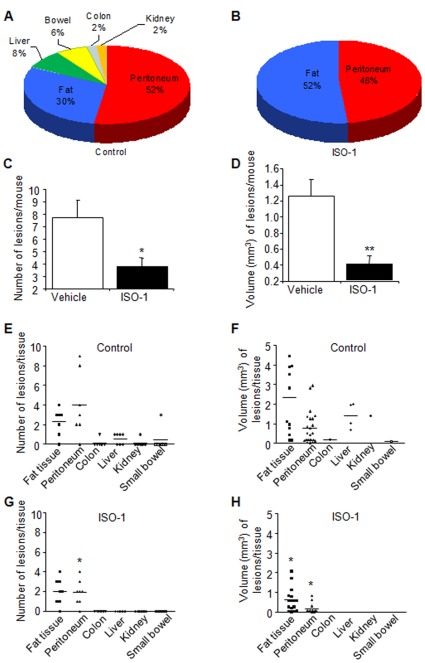
Effect of ISO-1 on endometrial implant number, volume and dissemination. Endometrial implants were localized using fluorescence stereomicroscopy, then counted and measured *in situ.* A and B) Schematic representation of colonized mouse organs or tissues (%). C and D) number and volume of lesions/mouse following treatment with vehicle (control) or 4 mg/kg ISO-1. E and F) Number and volume of lesions according the implantation site in mice treated with vehicle (control). G and H) Number and volume of lesions according the implantation site in mice treated with or 4 mg/kg ISO-1. Results were from 8 control mice treated with vehicle and 7 mice treated with ISO-1 (4 mg/kg). Data are mean ± SEM. *, **, P<0.05 and P<0.01, respectively, as compared to the control group.

Analysis of harvested lesions relative to the implantation site showed several invaded organs and tissues in control mice, such as the peritoneum (52%), fat tissue (30%), the liver (8%), the bowel (6%), the colon (2%) and the kidney (2%), while in mice treated with ISO-1, endometrial implants were only recovered in the peritoneum (48%) and fat tissue (52%) (Figure 4, A and B). Statistical analysis showed a significant decrease in lesion number and volume in ISO-1-treated mice compared with vehicle-treated controls (P<0.05 and P<0.01, respectively) (Figure 4, C and D). Furthermore, in mice treated with ISO-1, lesion number was significantly reduced in the peritoneum (P<0.05), while lesion size was reduced both in the peritoneum and fat tissue (P<0.05) (Figure 4E-H).

**Figure 5:**
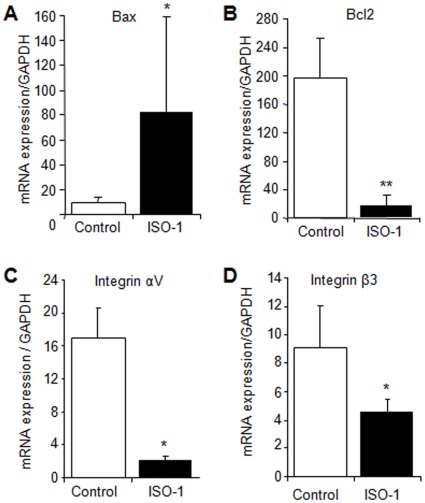
Real time PCR analysis of the expression of Bax, Bcl2, integrin αv and integrin β3 in endometriotic lesions. Lesions were harvested from mice treated with vehicle (control) or 4 mg/kg ISO-1. mRNA levels were normalized to that of the house-keeping gene GAPDH. Results were from 8 control mice treated with vehicle and 7 mice treated with ISO-1. Data are mean ± SEM. *, **, P<0.05 and P<0.01, respectively, as compared to the control group.

In order for the endometrial tissue to implant and growth into the ectopic sites, a complex network of biological processes would take place, including cell survival, adhesion, invasion and angiogenesis. To investigate the molecular pathways that may underlie the inhibitory effect of ISO-1 treatment on ectopic endometrial tissue growth and dissemination observed *in vivo*, we further assessed the expression of main apoptosis, adhesion, proteolysis and angiogenesis mediators found to be significantly dysregulated in human endometriotic lesions. Data displayed in Figure 5, A and B, shows a significant effect of ISO-1 on Bax and Bcl2. In fact, while the expression Bax, a major pro-apoptotic factor, was upregulated in lesions harvested from ISO-1-treated mice (P<0.05), the expression of Bcl2, which is known for its anti-apoptotic properties, was significantly downregulated (P<0.01). ISO-1 treatment also led to a significant inhibition of the expression of adhesion receptors integrins αv and β3 in endometrial implants (P < 0.05) (Figure 5, C and D).

**Figure 6:**
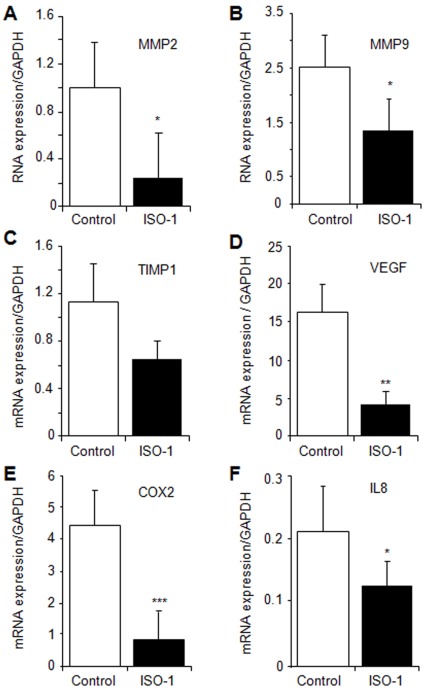
Real time PCR analysis of the expression of MMP2, MMP9, TIMP1, VEGF, COX2 and IL8 in endometriotic lesions. Lesions harvested from mice treated with vehicle (control) or 4 mg/kg ISO-1. mRNA levels were normalized to that of the house-keeping gene GAPDH. Results were from 8 control mice and 7 mice treated with ISO-1. Data are mean ± SEM. *, **, ***, P<0.05, P<0.01 and P<0.001, respectively, as compared to the control group.

We then analyzed the expression of MMP2 and MMP9, which have been found to be up-regulated in active endometriotic lesions and may contribute to the invasive capacity of endometrial implants as well as to angiogenesis [41], [42], [43]. As shown in Figure 6, A and B, treatment of mice with ISO-1 had a significant downregulatory effect on the expression of MMP2 and MMP9 (P<0.05). However, no statistically significant effect on TIMP1, a natural tissue inhibitor of MMPs, was noted (Figure 6C). Furthermore, treatment with ISO-1 significantly inhibited the expression of vascular endothelial cell growth factor (VEGF), a major angiogenic factor, (P<0.01) (Figure 6D) and that of COX2 and IL8 which play a role in endometriosis-associated inflammation, angiogenesis and tissue remodeling [28], [44], [45], [46], [47] (P<0.001 and P<0.05, respectively) (Figure 6, E and F).

## Discussion

It is well documented today that endometriosis is often associated with chronic pelvic inflammation. Significant immuno-inflammatory changes have been observed in the eutopic endometrium of women with endometriosis, but also in the peritoneal cavity of patients where the disease frequently develops, and within and around endometriotic lesions [1], [8], [9], [10], [11], [48] These changes may contribute to the development of the disease. For instance, immune cells, particularly macrophages, which are the main immune cell types found in the peritoneal fluid, seem to be less phagocytotic and despite their increased number and activation status, they lack the capability of eliminating endometrial debris [9], [49]. These cells resist apoptosis and produce proinflammatory, angiogenic, growth and tissue remodeling factors, which may contribute to the ectopic growth of endometrial tissue [12], [13], [14], [15], [16], [50]. Known for its capability to inhibit macrophage migration, MIF activates macrophages and may therefore play a role in retaining these cells into the inflammatory sites [23], [51]. Our previous studies showed an increased secretion of MIF by peritoneal macrophages of women with endometriosis [12] and further revealed an increased expression of this factor in eutopic endometrium and initial, active and vascularized endometriotic lesions [17]. Moreover, either local peritoneal fluid or systemic circulating levels of MIF were found to be higher in women with endometriosis and appeared to depend on the disease’s stage and major clinical symptoms (pain and infertility) [11], [17], [18], [19]. These observations were confirmed by other studies [20], [52]. Interestingly, according to Seeber et al, the association of MIF with cancer antigen (CA)-125, monocyte chemotactic protein 1 (MCP1) and leptin, can diagnose endometriosis in 48% of patients with a specificity equal to 93% [53]. Taken together, these findings strongly suggest that targeting MIF, using specific inhibitors, may represent an interesting strategy for treating endometriosis.

The present study used an *in vivo* model of experimentally induced endometriosis, where human endometrial tissue was allowed to implant and grow prior to any treatment. Both surface and deep endometrial implants were found, and the implanted tissue displayed endometrial stromal and glandular structures similar to those of the initial tissue. Implants were precisely identified, measured and harvested as they were labeled with a fluorescent marker before being inoculated into animals. Our data showed that ISO-1, a specific inhibitor of MIF [54], inhibits human endometrial tissue growth in the peritoneal cavity of mice and significantly reduces tissue size, number and extent of dissemination. ISO-1 treatment disrupted endometrial tissue structures and gland secretory activity, reduced tissue invasion and growth into the main colonized sites, such as the peritoneum and fat tissue, and abolished its development in the liver, the bowel, the colon and the kidney. Molecular analyses further showed that ISO-1 acted on several biological pathways closely associated with the pathogenesis of endometriosis. Actually, ISO-1 appeared to downregulate the expression of Bcl2 and to up-regulate in parallel the expression of Bax, which, respectively, favor cell survival and apoptosis [55]. Also, ISO-1 treatment significantly downregulated the expression of the proteolytic enzymes MMP2 and MMP9, but had no significant effect on their natural specific tissue inhibitor TIMP1 [42], [43]. These data suggest that ISO-1 treatment acts by altering the balance of pro- and anti-apoptotic factors in endometriotic implants and that of pro- and anti-proteolysis molecules. Such a dual complementary effect may, in combination with the significant inhibition of main adhesion, angiogenic and proinflammatory mediators such as integrins αv and β3, VEGF, IL8 and COX2, deeply affect endometrial tissue survival in the ectopic sites and reduce its growth, invasiveness and dissemination.

The development of endometriotic lesions requires critical steps including the capacity of the migrating endometrial tissue to survive and resist apoptosis, attach to and invade the host tissue, proliferate and activate the host angiogenic responses. This is supported by the detection of numerous alterations in the apoptotic pathways, including a significant up-regulation of the anti-apoptotic molecule Bcl2 and a significant down-regulation of the pro-apoptotic factor Bax [55], and an increased expression of integrin receptors αv and β3 which are also markers of active neovascularization and play an important role in cell adhesion to the extracellular matrix (ECM) [56], [57]. Other studies showed that MMPs, which are required for ECM degradation during tissue invasion 9,58 and crucial for sprouting of capillaries from pre-existing vessels, were over-expressed in active endometriotic lesions [41], [42], [43]. This was also reported for VEGF, IL8 and COX2, which are key mediators of angiogenesis, cell proliferation and inflammation [28], [44], [45], [46], [47], [59].

MIF is now known for being a multifunctional factor with a wide spectrum of effects and cell targets. MIF plays an essential role in tumorigenesis, tissue remodeling and angiogenesis [26], [27], [28], [29], [30], [31], [34], [35]. Recent data from the literature and our laboratory showed an important role for MIF in cell proliferation [60], [61], inhibition of apoptosis [62], [63], [64], stimulation of metalloproteinases [65], [66] and induction of angiogenesis [30], [31], [67], [68], [69], [70]. MIF stimulates COX2 expression in ectopic endometrial cells and elicit a pro-angiogenic and pro-inflammatory phenotype [28], [37], thereby potentiating their capability to stimulate the host angiogenic response and exacerbate the immuno-inflammatory reaction occurring in the implantation site.

ISO-1 binds to the enzymatic active site of MIF, thereby inhibiting its effects on target cells [38], [71]. MIF has two receptors CD74 and CD44, and the available literature including ours indicates that MIF activates cells via CD74 and triggers the activation of p38 and ERK 1, 2 MAPK signaling pathways [28], [37], [72]. Our previous studies with endometriotic cells showed the involvement of p38 and ERK pathways in VEGF mRNA synthesis and protein secretion and the inhibitory effect of ISO-1[37]. We also showed the involvement of these pathways in MIF-induced COX2 expression (mRNA and protein) and inhibition by ISO-1 [28]. Research is underway to assess whether these pathways are also involved in the expression of the MIF-induced or -inhibited factors shown in the present *in vivo* work, using primary endometriotic cells, but also endometrial cells from women with and without endometriosis, either at the transcriptional or the post-transcriptional level.

Nude mice are deficient in T cells, but still have the capability of mounting a partial immune response because of functional natural killer cells and macrophages [73], which may be activated by exogenous endometrial tissue. It was also shown that CD45 positive leukocytes are recruited into endometriotic lesions in nude mice [74]. However, further studies will also be necessary to evaluate the effects of ISO-1 with endometrial tissue from women with endometriosis since it differs from normal endometrium and showed an increased expression of MIF [17].

Several previous studies showed the benefit of targeting MIF for treating inflammatory diseases such as asthma, sepsis, [32], and viral infection [33]. Therefore, our data demonstrating for the first time that ISO-1, a specific antagonist of MIF, effectively interferes with the growth and progression of established endometriosis lesions *in vivo*, supports a potential therapeutic interest for this molecule. The current medical treatment of the disease is mainly based on the induction of a hypoestrogenic state, which, however, is associated with serious side effects and a high recurrence, and there is a crucial need for non-steroid targeted treatment of endometriosis.
